# Monthly Digest

**Published:** 1896-12

**Authors:** J. M. Walker


### MONTHLY DIGEST.

Reported for the DENTAL REGISTER by Mrs. J. M. Walker-

PLASTIC.*

DR. WM. B. AMES, in a paper read before the Chicago Dental Society, considered the oxyphosphates as materials worthy of the best efforts in conscientious and thorough work.

Experience has convinced him that by a proper selection of materials a good cement filling very rarely loses any of its substance when protected from friction, and that he can practice dentistry conscientiously by trusting to materials of this nature in a very liberal percentage of cases, viz.: in the teeth of all children, and of most young persons fairly-well matured, for young mothers, or young wives about to become mothers and for all small cavities on surfacess difficult of access. For large cavities requiring bold contour he inserted gold or porcelain inlays, set with oxyphosphate.

For large cavities on the anterior or posterior surfaces of second or third molars, and also for shallow grooves about the gum margins of the posterior teeth, he uses oxyphosphate of copper because of its ready adhesion to a plane surface, even if a trace of moisture is present.

He considers that the presence of soluble alkaline phosphates the cause, detracting most from the integrity of cements, the absence of alkaline adulteration of the phosphoric acid not only lending integrity to the mass but obviating the objectionable stickiness and leathery consistency of some cements, causing failure even with most conscientious effort to obtain the greatest good possible from the material.

The oxyposphates which he has found most free from the soluble alkaline phosphates as Eisfelder's, globe, concrete, Harvard, Poulson's, Dirigo and the two furnished by Justi.

He advises the use of a matrix wherever possible, and keep-

* Dental Review, March, 1895.

ing the material protected from the saliva as long as the exigencies of the case will permit.

The paper was discussed at some length, the profession being congratulated by Dr. E. Noyes upon the definite and important progress made in the manufacture of plastic cements. The solution of the questions of reliability and durability is all that is needed to revolutionize the use of filling materials as regards human teeth.

Dr. Fernandez secures the exact shade required in a cement by intermixing finely-pulverized jeweler's enamel, also burnishing this powder into the surface of a cement, filling before it is perfectly hard, prolonging insolubility. He uses gold matrices and gilt or nickel-plated spatulas, exclusively, in cement work.

Dr. J. W. Wassal burnishes the surface of oxyphosphate fillings, thereby giving a harder surface than by any other manipulation.

Dr. M. G. Jemison, in a paper read before the Minneapolis Dental Society,* reviews the records of history along the line of

ANÆSTHESIA,

With the modus-operandi of the agents most generally employed, including hypnotism and rapid breathing.

In the discussion Dr. W. X. Sudduth spoke of the value of hypnotic suggestions in connection with drug anæsthesia, the psychical aspect of the question being, to his mind, even more important than the physiological effect or the potentiality of the drug, the personality of the operator playing a very important part.

Dr. O. J. Oakey gave a resume of the history of Anæsthesia before the Hayden Dental Society. Dr. "J. W. R.," in the same journal† relates an incident showing the potency of cocaine, and the importance of having all medicine vials, both bottles and stoppers, carefully labeled. In the case mentioned a fistulous abscess was under treatment. At the first sitting $H_2 O_2$, followed by an essential oil, was injected through the tooth and

* Dental Review, March, 1895.

† Dental Review, April, 1895.

fistula. At the next sitting the injection was repeated, but no effervesence following the supposed $H_2$ $O_2$ it was found that cocaine 4 percent had been used, the similar vials being labeled only on the stopper the latter having been accidentally exchanged. The result was extensive inflammation and necrosis of a large plate of alveolar process. Though special pains were taken antiseptically, in removing the sequestrum, etc., the suture continued to slough for a considerable time, the vitality of the tissues being lowered by the cocaine. There were no toxic symptoms.

DR. O. A. WEISS (Minneapolis Dental Society)* reviewed briefly the various agents employed for producing

LOCAL ANÆSTHESIA, as refrigeration, electricity, and various drugs, especially cocaine, of which the paper is an exposition—considering the dose, the preparation, and the correctives. The infiltration method of Dr. Cholewa (Berlin) is given, as follows: A one-tenth-percent solution of cocaine in from five to ten parts of a two-tenths-percent solution of common salt is used, more than twenty fluid ounces being required for one grain of cocaine. It is used intracutaneously instead of subcutaneously. Anæsthesia is produced by infiltration, the sodium chloride assisting in the permeation of the tissues, so little cocaine being used that there is no risk of dangerous absorption. Doctor Weiss considers the physiological action of cocaine, the purposes for which it may be used, how to use it, upon whom to use it, and the antidotes and resuscitants.

DR. ROBERT MARSTON (England) † discusses the question, "Is the Anæsthesia Caused by Nitrous Oxid Merely Venous Coma?" reaching the conclusion that the process is essentially one of asphyxiation, the intermediate stage accepted as anæsthesia being simply a state of venous or asphyxial coma caused by the toxication of venous blood on the nervous centers. The physiological effects are incompatible with the supposition that it is an anæsthetic; appearances all distinguish it as a negative asphyxiant—an inert atmosphere which serves only to fill the dilating lungs during respiratory efforts, enabling the mechanical

* Dental Review, March, 1895.
† Items of interests, March-April, 1895.

performance of the respiratory mechanism during the temporary cessation of pulmonary chemical action. The absence of oxygen causes an accumulation of natural products, whose eagerness for the missing element increases with the increasing carbonic plethora, the toxic power of which, on the nerves, while obtunding their sensibility, increases their involuntary motor impulses and converges the vital energies to the point of assimilating an element which nature supplies abundantly on every side.

To prolong the effects of nitrous oxid, he would first administer a few whiffs of chloroform—not sufficient to produce a perceptible effect, but with the result of prolonging the anæsthetic period.

DR. P. ROSENBERG described, before the Medical Society,* a new method of

COMBINED ANÆSTHESIA, by which the lethal action of chloroform on the heart is entirely prevented. The mucous membrane of the nose is to be previously anesthetized by a spray of cocaine, preventing the reflex disturbance which is transmitted to the nerves, paralyzing the heart and respiration. Cocaine is an antidote to chloroform. The anæsthesia is more readily induced, and is free from after-effects, the patients waking rapidly with none of the usual discomforts.

### ETHYL BROMIDE AS A GENERAL ANÆSTHETIC IN DENTAL PRACTICE.†

GEORGE MORGENTHAL, M. D., shows the advantages which ethyl bromide possesses over nitrous oxide gas, ether and chloroform as an anæsthetic agent, in that it does not produce any change in the red blood corpuscles, causes no after-sickness, has no unpleasant odor, creates no irritation of the mucous membrane, is rapidly eliminated from the body by the lungs. Recovery is very rapid, the patient being able to walk alone after a few moments, and to leave the office within from fifteen to thirty minutes, feeling bright and cheerful. He commends it as worthy of trial in suitable cases, from its manifold and obvious advantages.

* Dental Review, March, 1895.

† Dental Review, March, 1895.

CONSOLIDATION OF IMPLANTED TEETH.

Dr. Oscar Arnold (Paris),* contributes the following points toward the solution of the question of the mode of retention of implanted teeth. Having come into possession of an inferior maxilla having "a second milk molar ankylosed between the first permanent molar and the first bicuspid," he sawed the maxilla in two in search for the missing second bicuspid. He found no trace of this tooth. All over the surface of the root of the ankylosed deciduous tooth the pericementum had disappeared, the cementum was destroyed in large part and substituted by an osseous proliferation coming from the alveolar wall, the pulp chamber being filled with secondary dentine. Sections made for microscopic study show such "intimate connection between cementum and osseous tissue that it is impossible to say where bone begins and the root ends."

To explain the pathological process he thinks that the deciduous tooth not being shed became an obstacle to the normal development of the bicuspid, which, being unable to force its way through the maxilla, created an irritation and over-excitement of activity in that region, causing absorption both of the developing tooth and of part of the roots of the milk-tooth. The cause of the irritation being thus removed, compact bone-tissue filled the hollow left by the second bicuspid and the bays on the surfaces of the roots of the deciduous molar. This he concludes must be the process that occurs between the bone and the root of an implanted tooth, the latter acting as a foreign body irritating the bone and producing an ostitis, with formation of lymph-cells which form an embryonal membrane throughout the periphery of the alveolus. That these embryonal cells advance toward the root until they cover it, then decalcification and erosion of the root begin, giant cells with acid reaction forming little bays on the surface of the root; conditions being good (the implanted tooth firmly fixed in position) resorption stops, the embryonal tissue is vascularized, calcic deposits are formed, until finally the root and alveolar walls are, as it were, soldered together.

As a practical deduction, Dr. Amoedo decalcifies the cemen-

* Cosmos, March, 1895.

tum of a tooth to be implanted to the depth of half a millimeter, and ligates it to secure immobility.*

EX-SECTION OF THE INFERIOR DENTAL NERVE.

Dr. T. W. Brophy, in a paper read before the Chicago Dental Society,† describes his new method as follows: With a long, flexible, graduated drill, similar to the Gates Dental Canal Drill; the inferior canal is entered at its mental foramen, and the drill carried backward to the inferior dental foramen, thoroughly removing the contents of the canal, the drill being sufficiently flexible to follow the upward curve of the canal; if necessary, the drill may be followed by a broach of large form modeled after the Donaldson Canal Broach, to remove any remnants of nerve tissue. The after-treatment is simply anti-septic cleanliness.

† Dental Review, March, 1895.

